# Obstructing Pancreatic Ductal Calculus: A Case Report and Literature Review

**DOI:** 10.7759/cureus.7730

**Published:** 2020-04-18

**Authors:** Zeid Nesheiwat, Taha Sheikh, Dipen Patel, Cameron Burmeister, Mamtha Balla

**Affiliations:** 1 Cardiology, University of Toledo Medical Center, Toledo, USA; 2 Internal Medicine, University of Toledo College of Medicine and Life Sciences, Toledo, USA; 3 Internal Medicine, University of Toledo Medical Center, Toledo, USA; 4 Internal Medicine, University of Toledo, Toledo, USA; 5 Internal Medicine, Promedical Toledo Hospital, Toledo, USA

**Keywords:** calculus, pancreas, shock wave lithotripsy, endoscopic retrograde cholangiopancreatography, eswl, pancreatolithiasis, chronic pancreatitis, ercp

## Abstract

Pancreatic calculi are typically a sequela of chronic pancreatitis. Here, we present a patient who was found to have an obstructing one-centimeter pancreatic calculus secondary to recurrent gallstone pancreatitis. Recent retrospective studies have focused on the optimal treatment of large pancreatic calculi that were defined as greater than five millimeters. But most studies fail to comment on much larger stone as in this case report. Further guidelines and investigation need to be done aiming toward the optimal treatment of relatively large pancreatic stones.

## Introduction

Pancreatic calculi (PC), also known as pancreatolithiasis, is a late complication of chronic pancreatitis (CP) [1]. Pancreatolithiasis occurs in CP irrespective of the cause of chronic pancreatitis. Repeated inflammation, subsequent fibrosis, stasis of pancreatic secretions, and subsequent saponification leads to nidus formation within the pancreatic ducts and parenchyma. Calcium, which is abundant in pancreatic juice, is regulated by bicarbonate (HCO3-), citrate, and pancreatic stone protein (PSP). Due to parenchymal destruction, these regulatory mechanisms are deranged in chronic pancreatitis, allowing for precipitation of calcium in the form of stones [2]. Over time, calcium carbonate deposits in layers over the nidus with each attack of acute on chronic pancreatitis, adding to the enlarging size of the nidus until it starts to obstruct the pancreatic ducts. The nidus may form anywhere in the ductal system or even in the parenchyma, the latter being smaller and easier to treat via endoscopic retrograde cholangiopancreatography (ERCP). These calculi lead to outflow obstruction, subsequent ductal hypertension, then eventual ischemia [2, 3]. This results in precipitation of more episodes of pancreatitis and characteristic pain, propagating a feedforward cycle of chronic pancreatitis unless intervention is performed to relieve the obstruction. In addition, hypercalcemia may cause a rise in the level of calcium in pancreatic juice, which accelerates the formation of pancreatic stones in patients with hyperparathyroidism [2]. We present a case of a relatively large, one-centimeter ductal pancreatolithiasis formed in the setting of recurrent gallstone pancreatitis. Optimal management of this size of stone has not been investigated. 

## Case presentation

An 89-year-old woman with a past medical history of coronary artery disease status post triple-vessel coronary artery bypass graft, heart failure with preserved ejection fraction, atrial fibrillation on warfarin, breast cancer status post left mastectomy, chronic obstructive pulmonary disease, cholelithiasis status post cholecystectomy and moderate aortic stenosis who presented to our institution with a chief complaint of epigastric pain and weight loss of one-month duration that was steadily worsening over the past two weeks. On initial examination, the patient was hemodynamically stable and initial lab work revealed normal liver function tests, white blood cell count, lipase and an international normalized ratio (INR) of 3.3 (patient is on warfarin for her atrial fibrillation). 

**Figure 1:**
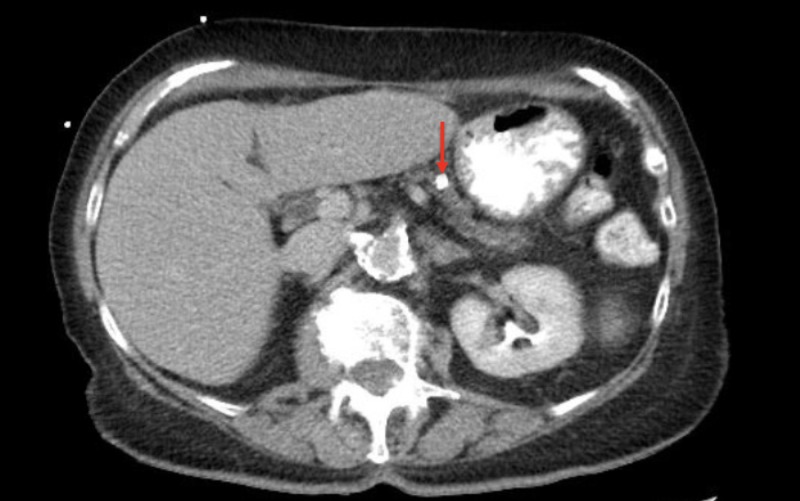
Computed tomography of the abdomen in transverse view showing a 10 mm pancreatic calculus (red arrow)

**Figure 2:**
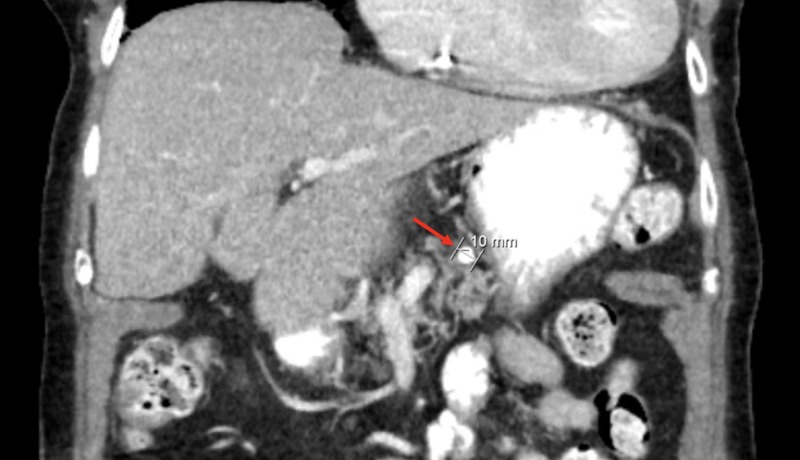
Computed tomography of the abdomen in coronal view showing a 10 mm pancreatic calculus (red arrow)

**Figure 3:**
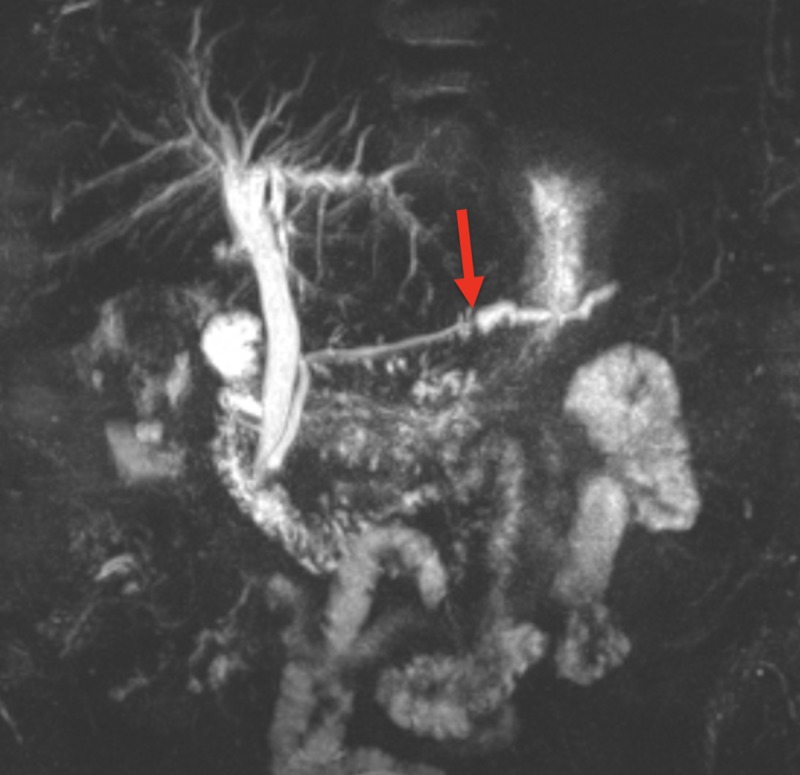
MRCP in coronal view showing an abrupt transition from a dilated distal pancreatic duct to a narrow more proximal pancreatic duct (red arrow) indicating the presence of a pancreatic stone MRCP - magnetic resonance cholangiopancreatography

**Figure 4:**
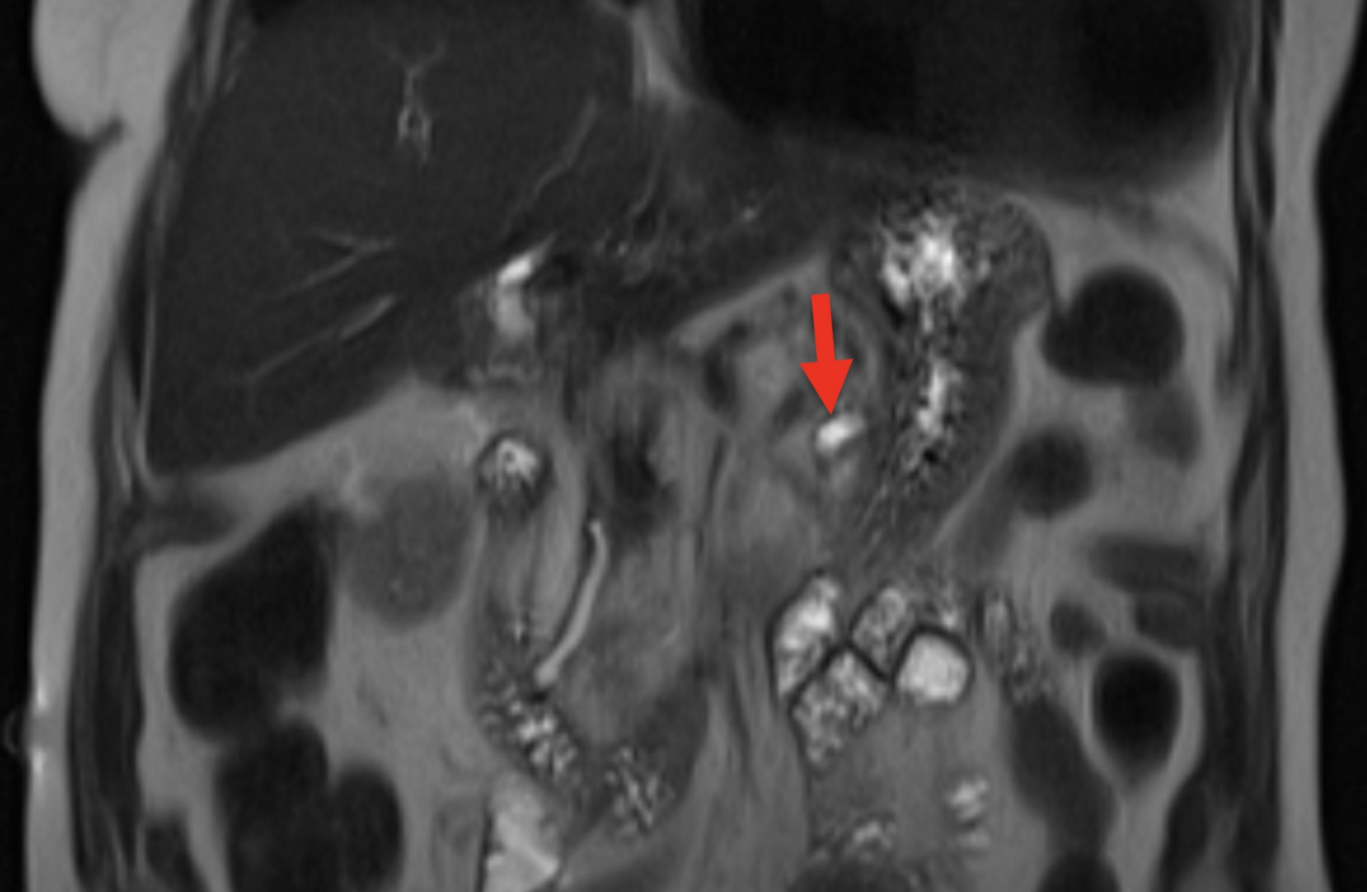
MRCP in coronal view showing a dilated pancreatic duct distal to the obstructing pancreatic stone (red arrow) MRCP - magnetic resonance cholangiopancreatography

**Figure 5:**
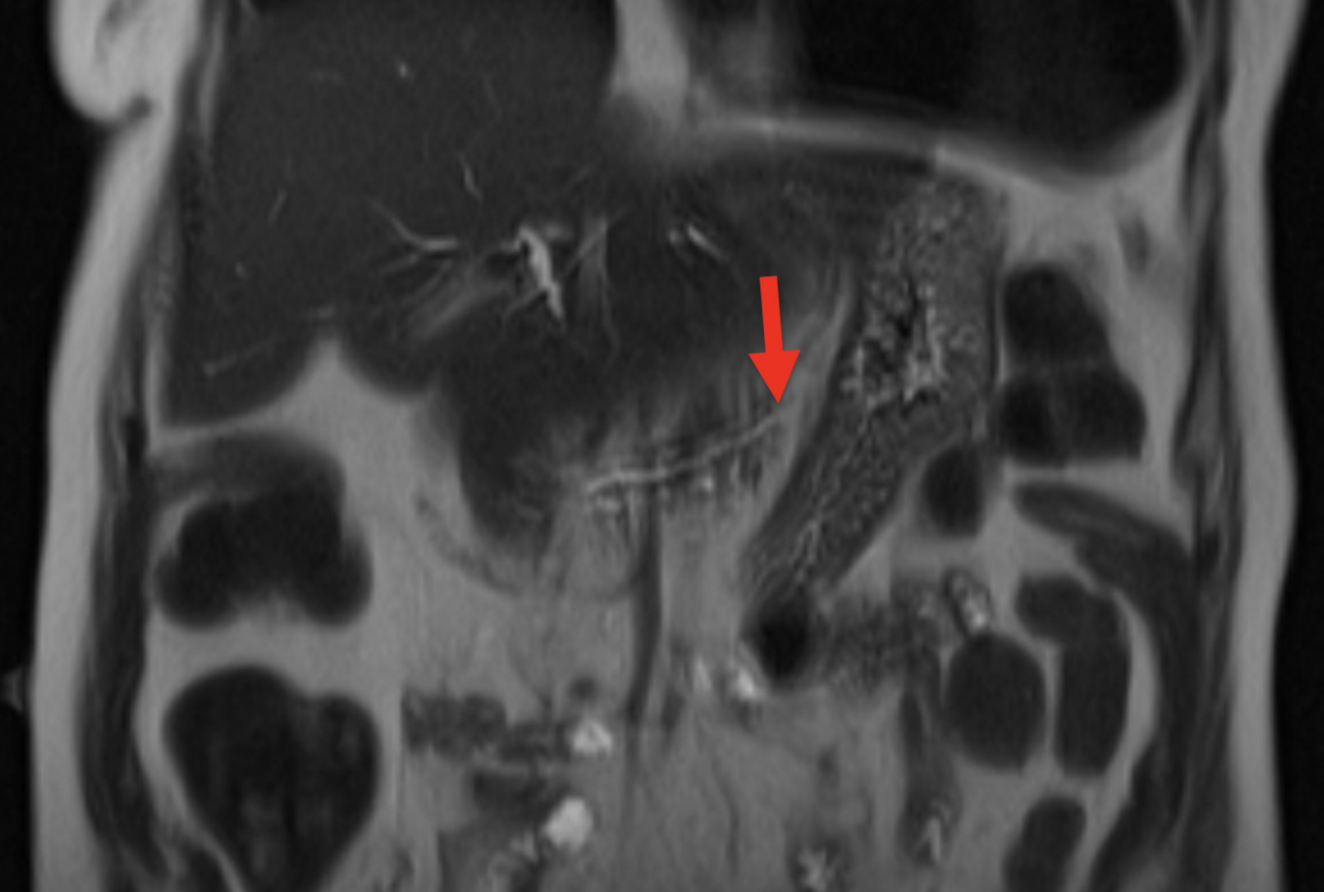
MRCP in coronal view showing a narrow pancreatic duct proximal to the obstructing pancreatic stone (red arrow) MRCP - magnetic resonance cholangiopancreatography

Computer tomography of the abdomen revealed dilation of the pancreatic duct with a one centimeter pancreatic ductal obstructing mass (Figures 1 and 2). The patient was admitted for further investigation. There was suspicion for possible neoplasm. Gastroenterology was consulted and magnetic resonance cholangiopancreatography was completed revealing pancreatic duct dilatation with an abrupt transition to a narrow duct indicating a large obstructive calculus (Figures 3-5). ERCP was then performed, after the reversal of INR, confirming a pancreatic stone in the body of the pancreas. The advanced endoscopist at that time tried to retrieve the stone but failed due to its large size. She was discharged and given strict instructions to follow up with gastroenterology for definitive management of her large pancreatic calculus by extracorporeal shock wave lithotripsy (ESWL) with ERCP. The patient’s pain was controlled prior to discharge. 

## Discussion

Treatments for pancreatic calculi include surgical, endoscopic techniques and ESWL. According to the European Society of Gastrointestinal Endoscopy (2015), the preferred treatment method involves an endoscopic approach with reasonably fewer complications as compared to an open approach; however, there are limitations of endoscopic techniques as success is usually only present with small stones and those located in the main pancreatic duct. Unusual presentations of pancreatolithiasis include stones in any duct besides the main pancreatic duct, as well as parenchymal pancreatolithiasis, which cause duct blockage by external compression. For unusual presentations of pancreatolithiasis and larger stones (>5mm), ESWL is recommended. Surgery is the last resort and typically used for incarcerated stones or significant pseudocyst formation [3, 4]. As shown by a case series, those with short term duration of pain have better clinical outcomes than those with long-standing more chronic disease [5]. 

Approximately 33% of patients with chronic pancreatitis develop pancreatic stones. Around 50% of patients who develop pancreatic stones do so in the main pancreatic duct. A stricture or tight sphincter of Oddi typically contributes to stone formation. Endoscopic management of these patients involves sphincterotomy, stricture dilation, and stone removal [5]. Stones larger than five millimeters are considered large and typically treated with ESWL to fragment them prior to endoscopic management [3, 6, 7]. A review of the current literature fails to identify a pancreatic stone more than 0.05 cm, such as in this case. Current treatments for painful pancreatic calculi in chronic pancreatitis are ERCP, medications, ESWL, surgery or some combination of the above. For the main pancreatic ductal stone, ESWL is typically successfully and calculi fragmentation by ESWL is successful in approximately 90% of cases. The European Society of Gastrointestinal Endoscopy recommends endoscopic therapy in the forms of ERCP and/or ESWL prior to surgical treatment for pancreatitis. This is primarily due to the invasive nature of the surgery and the higher mortality and morbidity associated with surgical outcomes in chronic pancreatitis [8]. Newer treatment modalities for pancreatic calculi are emerging, such as laser or electrohydraulic lithotripsy.

Studies show that patients benefit from stone clearance by having the relief of immediate pain. Researchers demonstrated that anywhere from 77% to 100% of patients had immediate pain relief in acute pancreatitis after partial or complete removal of pancreatic calculi [9-12]. These patients also benefit from less narcotic use due to decreased pain levels [7]. The formation of pancreatic calculi is a relatively common complication in patients with chronic pancreatitis. However, pancreatic calculi can present uncommonly, such as in the above case, which can lead to further workup, imaging and need for invasive procedures. Thus, pancreatic calculi should be included in the differential for a patient with a history of chronic pancreatitis who presents with acute abdominal pain.

## Conclusions

Pancreatic calculi should be included in the differential for a patient with a history of chronic pancreatitis who presents with acute abdominal pain. Pancreatic calculus, most notably, occurs secondary to chronic pancreatitis. An endoscopic approach is the preferred treatment modality versus the open surgical method given its fewer complications. We present a patient who was found to have a one centimeter obstructing pancreatic calculi secondary to gallstone pancreatitis. Current research is limited, primarily focusing on smaller stones (less than 0.5 mm). There is limited data regarding the optimal approach for larger stones as in this case. Further research needs to be done evaluating the optimal treatment of relatively large pancreatic stones.
